# Association between redox dysregulation and vulnerability to cognitive deficits induced by maternal immune activation

**DOI:** 10.1038/s41398-025-03398-0

**Published:** 2025-05-26

**Authors:** Francesca McEwan, Chiho Kambara, Jarred M. Lorusso, Michael K. Harte, Jocelyn D. Glazier, Reinmar Hager

**Affiliations:** 1 Division of Evolution, Infection, and Genomics, School of Biological Sciences, Manchester Academic Health Science Centre, Faculty of Biology, Medicine & Health, University of Manchester, Manchester, M13 9PL United Kingdom; 2 Division of Cell Matrix & Regenerative Medicine, School of Biological Sciences, Manchester Academic Health Science Centre, Faculty of Biology, Medicine & Health, University of Manchester, Manchester, M13 9PL United Kingdom; 3 School of Humanities and Social Science, University of Brighton, Brighton, BN2 4AT United Kingdom; 4 Division of Pharmacy & Optometry, School of Health Sciences, Geoffrey Jefferson Brain Research Centre, Faculty of Biology, Medicine & Health, University of Manchester, Manchester, M13 9PL United Kingdom

**Keywords:** Neuroscience, Molecular neuroscience

## Abstract

Exposure to maternal immune activation (MIA) *in utero* is a major risk factor for neurodevelopmental disorders, including schizophrenia. However, a proportion of individuals are resilient to developing schizophrenia following exposure to MIA, which has also been reported in animal models of MIA. The molecular mechanisms leading to resilient and vulnerable behavioural phenotypes remain poorly understood, and we currently lack reliable blood biomarkers that predict resilience or vulnerability. Redox dysregulation, caused by an imbalance between oxidative stress and antioxidant defence mechanisms, has recently been predicted to be central to the pathogenesis of schizophrenia. Here, we use a poly(I:C)-induced MIA model of schizophrenia to investigate mechanisms underlying cognitive dysfunction and redox dysregulation in resilient and vulnerable individuals. We show that activity of the antioxidant enzyme superoxide dismutase (SOD) was reduced in the plasma of poly(I:C) offspring with a cognitive deficit, in contrast to individuals with typical cognition during both adolescence and adulthood. However, SOD activity in the hippocampus was not significantly different between vulnerable and resilient offspring. In addition, the lipid peroxidation marker malondialdehyde (MDA) and the pro-inflammatory cytokine IL-6 were not differentially expressed within the hippocampus or plasma of vulnerable poly(I:C) offspring. Our results suggest that reduced plasma SOD activity may be a potential blood biomarker to identify resilience or vulnerability to MIA-induced cognitive deficits. Further research is necessary to determine if reduced antioxidant capacity is present in plasma prior to symptom presentation and to understand if this predicts redox dysregulation in the brain.

## Introduction

Neurodevelopmental disorders (NDDs), including schizophrenia (SCZ) and autism spectrum disorder, are prevalent and debilitating disorders, which have both genetic and environmental risk factors [1, 2]. While previous work has been particularly focused on investigating the genetic aetiology of NDDs, ample evidence suggests environmental exposures during critical periods of development play more of a fundamental role in the aetiology of NDDs than previously believed [3, 4].

Exposure to maternal immune activation (MIA) *in utero* has now been established as a major risk factor for SCZ in the offspring, as evidenced by epidemiological studies investigating naturally-occurring epidemics, such as the 1957 Influenza epidemic in Finland [5, 6], as well as birth cohort data where MIA is confirmed through serological samples [7, 8]. However, not all individuals exposed to MIA *in utero* will develop SCZ [9, 10]. Furthermore, animal models show a similar variability in response to MIA. When MIA is induced by administration of the viral mimetic poly(I:C) to the pregnant dam, a proportion of her offspring are “resilient” to developing behavioural impairments [11–14]. Therefore, identifying biomarkers that link causative molecular mechanisms and disease vulnerability following MIA is essential to predict those at risk of developing SCZ, with the ultimate objective being to prevent or better treat the clinical cause of the disorder [15].

Recently, a growing body of evidence suggests that redox dysregulation, an imbalance between oxidative stress and antioxidant mechanisms, may be central to the pathogenesis of SCZ. This can be caused by factors including neuroinflammation, mitochondrial dysfunction or NMDA receptor hypofunction, initiating oxidative stress and persistent neurological impairments [16]. Accordingly, a number of oxidative stress and pro-inflammatory blood biomarkers have been consistently associated with the disorder [17–19]. However, certain biomarkers may be altered in the acute phase of illness only, resolving with a reduction in symptoms [17]. Furthermore, antipsychotics directly alter peripheral inflammation and oxidative stress markers [20, 21]. It is therefore vital to differentiate between “state” or “trait” markers, defined as markers which are associated with the status of clinical manifestation of the disorder only, and markers that are consistently present, including prior to the onset of symptoms, respectively [22, 23]. The latter type would not only be more representative of causative mechanisms but may be used to predict SCZ outcome prior to diagnosis.

To date, few studies have investigated blood biomarkers within the MIA model [24] and, to our knowledge, no studies have assessed blood biomarkers following stratification of offspring into resilient and vulnerable subtypes based on behavioural output. However, a recent study used plasma cytokine profiles to cluster offspring into high and low cytokine status groups and showed that cytokine status was predictive of social cognition but not working memory phenotype [14]. While previous research has highlighted immune dysregulation in MIA offspring, a more comprehensive understanding of redox and inflammatory pathways underlying behavioural vulnerability remains limited. In this study, we investigate redox dysregulation in poly(I:C)-induced MIA offspring stratified into resilient and vulnerable subtypes based on novel object recognition (NOR) phenotype during both adolescence and adulthood. Specifically, we focus on oxidative stress markers such as superoxide dismutase (SOD) activity and malondialdehyde (MDA) levels, given their associations with cognition [25, 26] and more specifically novel object recognition [27–29].

Oxidative stress and inflammation have been proposed to be tightly interconnected in a self-amplifying cycle, where increased oxidative damage, regulated in part by SOD and marked by elevated MDA levels, triggers inflammatory responses, with chronic inflammation further disrupting redox balance, perpetuating cellular dysfunction [16, 30]. Therefore, investigating these markers alongside immune mediators may provide a more comprehensive understanding of the mechanisms underlying behavioural vulnerability in MIA offspring. IL-6 was chosen due to its essential role in mediating the MIA response [31, 32], but future studies may benefit from exploring additional inflammatory markers such as IL-1β and TNF-α to provide a more comprehensive picture of immune dysfunction in MIA-induced cognitive deficits.

## Methods

### Animals

Animal experiments were conducted in the Biological Service Facility (BSF) at The University of Manchester, and procedures were conducted under the authority of project licence PP6794596 in accordance with the Animals (Scientific Procedures) Act 1986. All work was approved by the University of Manchester Animal Welfare and Ethical Review Body. Wistar rats (Charles River, Germany) were housed in groups of 3–5 in individually ventilated cages (IVC; GR1800; Tecniplast, UK) with *ad libitum* access to standard rat chow (Special Diet Services, UK) and water. The room was maintained at a temperature of 21–23 °C and humidity of 55–60% on a 12 h light-dark cycle with lights on at 07:00 h.

### Poly(I:C)-induced MIA

Female rats were paired with male rats in a 1:1 ratio for overnight mating in an individually ventilated cage (IVC) containing a timed mating grid with a black card beneath a grid to facilitate identification of the vaginal plug. No bedding was provided in the cage during mating, as the presence of bedding would hinder the accurate observation of the vaginal plug. The observation of the vaginal plug was designated as gestational day (GD) 1. On GD15, dams were randomly allocated into two groups and administered with an intraperitoneal injection of low molecular weight poly(I:C) buffered with potassium salt (LOT: 5936-43-03; InvivoGen, France) in endotoxin-free 0.9% saline at 10 mg/kg body weight or endotoxin-free 0.9% saline (as a vehicle control). 3 h post-injection 0.5 mL blood was extracted from the lateral tail vein of dams and IL-6 concentration was measured, as described below. Dams administered with poly(I:C) showed elevated IL-6 concentration in contrast to those administered with saline, thus confirming maternal immune activation (Supplementary Figure 1a). The birth date of pups was designated postnatal day (PD) 0. On PD1, offspring were sexed by identifying anogenital distance differences and litters were culled to a size of ten, with an optimum of five males and five females per litter. Details of litter size, pup weight and sex ratios are included in Supplementary Figure 1. Animals were weaned on PD28 and housed in same-sex and treatment group cages.

### Novel object recognition (NOR) test

Animals were assessed in the NOR task, as described previously [13]. Separate animals were tested during adolescence (PD36-38) and adulthood (PD91-93). A NOR arena was used, which was a black polyvinyl square box with a white grid floor. Offspring were first habituated to the NOR arena for 20 min for two consecutive days. On day 3, offspring were then placed into the arena and were exposed to two identical novel objects and allowed to explore for 3 min (acquisition phase). They were then returned to their home cage for a 2 min inter-trial interval (ITI), and the arena and objects were cleaned with 70% ethanol. The objects were then replaced with one object identical to that in the acquisition phase (familiar object) and one completely novel object. Rats were then placed back into the arena and left to explore the objects for another 3 min (retention phase). A discrimination index (DI) was used to compare NOR memory performance between groups during the retention phase:

$$Discrimination\,index(DI)=\frac{novel\,item\,exploration\,(s)-familiar\,item\,exploration(s)}{novel\,item\,exploration\,(s)+familiar\,item\,exploration(s)}$$

DI scores can range from +1 to −1, with a positive score reflecting a novelty preference and a negative score indicating that more time was spent exploring the familiar item. A score close to 0 indicates no preference for the novel or familiar item and hence, a NOR memory deficit [33].

The test was scored manually by one rater who was blinded to the experimental groups. To ensure reliability, a second rater independently confirmed DI scores by re-scoring a subset of randomised videos. Prior to scoring the retention phase, a number of validations were performed. First, we showed that there was no effect of group or sex on preference towards left or right objects at either developmental stage during the acquisition phase (Supplementary Figure 2a). Additionally, total object exploration during the acquisition or retention phase was not predicted by group or sex (Supplementary Figure 2c, d).

### Offspring plasma and brain collection

On PD42 (adolescence) and PD98 (adulthood), offspring were killed by asphyxiation with CO_2_ (2 L/min) followed by permanent cessation of circulation. Blood was collected from the femoral artery and transferred to an EDTA-coated micro sample tube (Sarstedt, Germany; 41.3395.005), which was centrifuged at 14,000 x g for 1 min and the plasma was collected and stored at −80 °C. Brains were removed, immediately flash-frozen using dry ice, and stored at −80 °C.

### Tissue processing

Brains were dissected whilst frozen on dry ice. The brain was first divided into two hemispheres and left or right hemispheres were selected at random from each individual for downstream processing. The dorsal hippocampus was then removed using the Rat Brain Atlas as a guide [34]. Proteins were extracted using the Nuclear Extract Kit (40010; Active Motif, Germany) according to the manufacturer’s instructions. Briefly, samples were homogenised in hypotonic buffer using a pestle. Following incubation on ice and centrifugation at 850 x g for 10 min at 4 °C, the supernatant (matrix) was removed and the pellet, which contained a single-cell slurry, was resuspended in hypotonic buffer, followed by detergent, to lyse the cells. Centrifugation was then performed at 14,000 x g for 30 s at 4 °C. The cytoplasmic fraction (supernatant) was then removed and added to the matrix fraction to form the post-nuclear supernatant (PNS). The protein concentration of the PNS fraction was quantified using the Bradford protein assay (Bio-Rad, USA; [35]), according to the manufacturer’s instructions.

### Oxidative stress and cytokine assays

For superoxide dismutase (SOD) activity measurement, the EpiQuik Superoxide Dismutase Activity/Inhibition Assay Kit (OP-0001; Epigentek, USA) was used according to the manufacturer’s instructions, using a standard curve ranging from 0.0078 – 0.5 U/µL. Plasma input was 30 µL and dorsal hippocampal PNS input was 30 µg, diluted in 30 µL SD1.

For malondialdehyde (MDA) measurement, the CheKine™ Micro Lipid Peroxidation Assay kit was used (KTB1050-EN; Abbkine, USA) according to manufacturer’s instructions. In brief, 100 µL plasma sample, 50 µg dorsal hippocampal PNS diluted in 100 µL dH_2_O or 100 µL dH_2_O for the blank, was added to 300 µL reaction mix and heated at 95 °C for 30 min. The samples were then cooled before being centrifuged at 10,000 x g for 10 min, and supernatant absorbance was measured and calculated relative to the blank.

For IL-6, we used the DuoSet ELISA (DY008; R&D systems, UK) following the manufacturer’s instructions, with a standard curve ranging from 7.813 – 2000 pg/mL.

### Statistical analyses

To estimate required sample sizes, we used G*Power statistical software (v3.1, Germany; Faul et al., 2009). Effect sizes, comparing treatment poly(I:C) to vehicle, were calculated based on our previous work within the group, indicating a medium to large effect size (f = 0.25–0.4) [13, 36, 37]. Thus, a minimum of six offspring/sex/group provides sufficient power of (1-β) =0.8 and a type 1 error rate (α) of 0.05 for molecular analyses. However, behavioural data was collected from 11–13 offspring/sex/group/developmental stage due to the lower effect size of behavioural traits. The total dam number was eight per group (poly(I:C) and vehicle), with only one or two offspring/sex/developmental stage used from the same dam.

All statistical analyses were conducted using IBM SPSS Statistics for Windows (v28.0; SPSS Inc, USA) and figures were created using GraphPad Prism (v9.0; GraphPad, USA). To assess whether treatment group, sex or cluster membership affected offspring outcomes, we used a general linear mixed model (GLMM) with dam as a random factor to account for differences caused by the pre- and postnatal environment, shared by individuals from the same dam [38, 39].

A two-step clustering method was used [13, 14] to group offspring into typical and deficit memory performance groups using NOR DI as behavioural input. This uses a log likelihood measure to determine the probability of cluster membership and determines the number of clusters using the Bayesian Information Criterion. Only clusters which were over 0.5 silhouette measure of cohesion and separation were accepted [40, 41]. The Chi-squared test was used to determine whether treatment group or sex affected distribution across clusters. Resilient poly(I:C) offspring were individuals clustered in the “typical” memory group (CL1), whereas vulnerable poly(I:C) offspring were clustered into the “deficit” memory group (CL2).

## Results

### MIA offspring exhibit reduced performance in NOR and can be clustered into resilient and vulnerable groups

We first sought to determine if poly(I:C) offspring exhibited a deficit in NOR and if this behavioural output could be used to cluster offspring according to resilience or vulnerability to poly(I:C)-induced MIA.

**Fig. 1:**
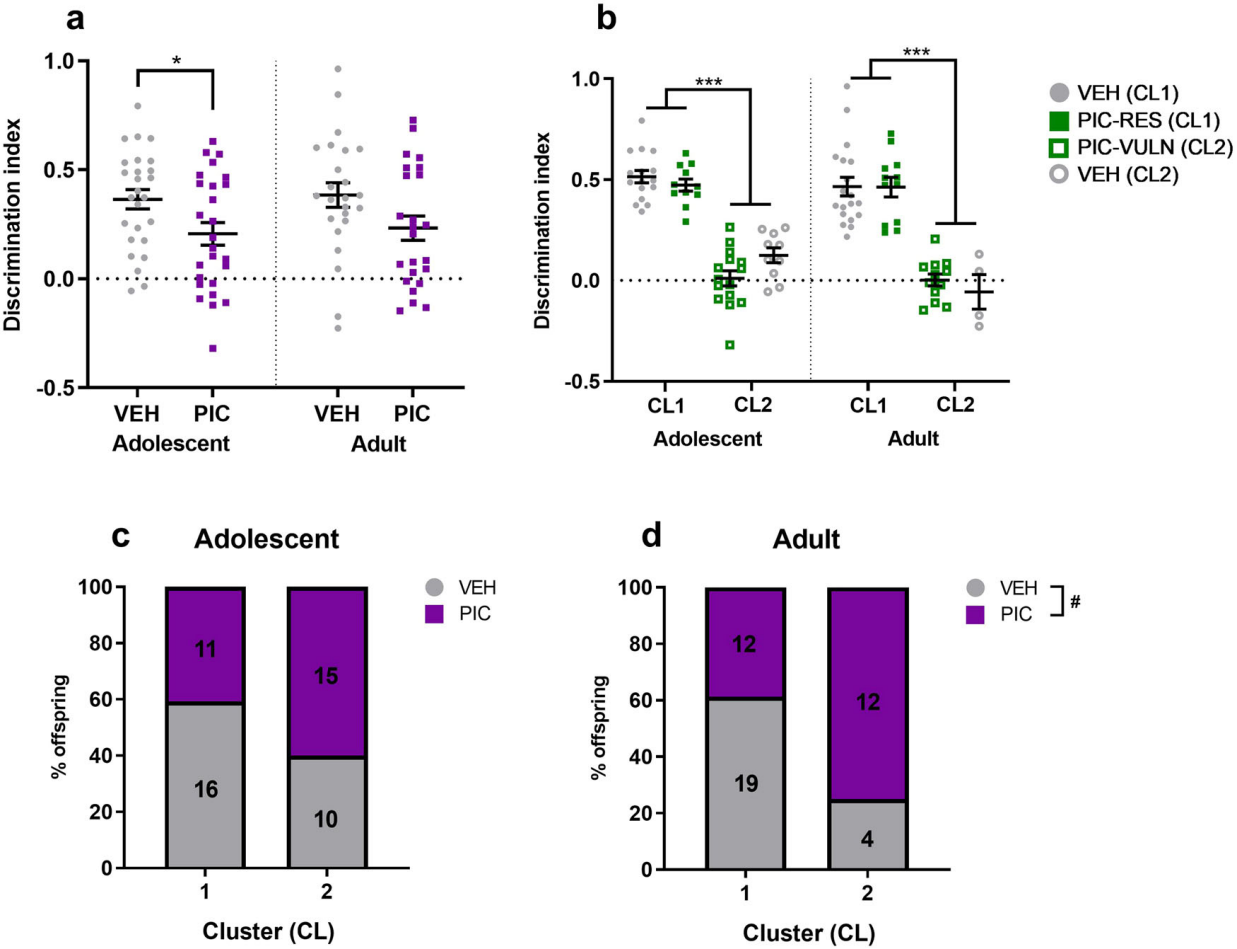
MIA offspring exhibit reduced performance in NOR and can be clustered into resilient and vulnerable groups. **a** NOR performance when measured by discrimination index during adolescence and adulthood (*n* = 23–26/group/age). **b** Discrimination index according to cluster. **c** Cluster distribution during adolescence where y axis represents percentage of offspring in each cluster, number on bar describes number of offspring in cluster. **d** Cluster distribution during adulthood where y axis represents percentage of offspring in each cluster, number on bar describes number of offspring in cluster. CL1=typical memory, CL2=deficit memory, PIC=poly(I:C), PIC-RES=resilient poly(I:C) offspring (CL1), PIC-VULN=vulnerable poly(I:C) offspring (CL2), VEH=all vehicle offspring, VEH (CL1)=vehicle offspring belonging to typical memory cluster, VEH (CL2)=vehicle offspring belonging to deficit cluster. *p < 0.05, ***p < 0.001. #*p* < 0.05 in Chi-squared test. Data are presented as mean ± SEM.

NOR performance, measured as DI, was significantly impaired in poly(I:C) offspring during adolescence (Fig. 1a; F_1,50_ = 5.395, *p* = 0.024). However, performance was not significantly different during adulthood (Fig. 1a; F_1,13.76_ = 2.056, *p* = 0.174). Furthermore, there was no main effect of sex or group on raw exploratory times of the novel and familiar object during adolescence or adulthood (Supplementary Figure 2b).

NOR DI was then used to cluster offspring into resilient and vulnerable subtypes. While a proportion of poly(I:C) offspring did not show a deficit (CL1), more poly(I:C) offspring belonged to the deficit NOR performance cluster (CL2) during both adolescence and adulthood (Fig. 1c, d). Similarly, a subset of vehicle offspring showed a deficit (CL2). While more vehicle offspring belonged to the typical (CL1) cluster at both ages, a larger proportion of vehicle offspring could not perform the task during adolescence. Thus, treatment group only significantly affected the distribution during adulthood (Fig. 1d; Χ^2^ = 5.562, *p* = 0.019).

There was no effect of sex on cluster distribution. Figure 1b shows NOR performance according to clusters. The DI of CL2 was significantly reduced in contrast to CL1 during both adolescence (F_1,41.41_ = 164.657, *p* < 0.001) and adulthood (F_1,45_ = 164.657, *p* < 0.001). In agreement with previous data [14], poly(I:C) offspring exhibited within-litter variability, with each litter containing a proportion of offspring clustered into both CL1 and CL2 (Supplementary Figure 3).

### Resilience to maternal immune activation is predicted by activity of the antioxidant SOD within the plasma

**Fig. 2:**
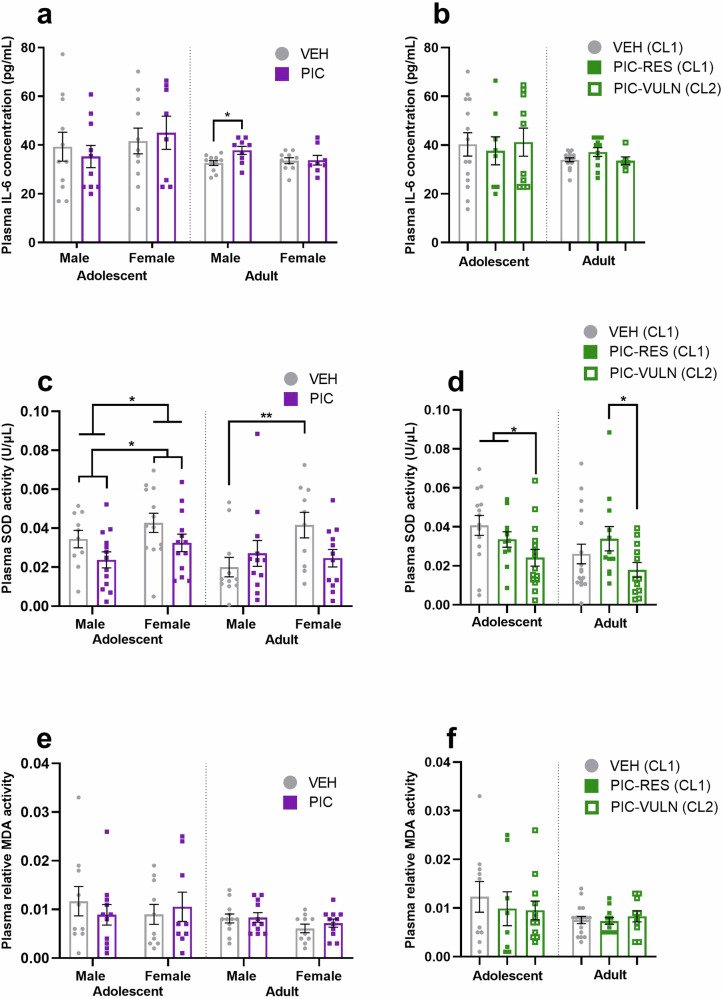
Offspring plasma biomarkers according to treatment and cluster membership during adolescence and adulthood. **a** IL-6 concentration according to treatment group (*n* = 8–11/sex/group/age). **b** IL-6 concentration according to cluster (*n* = 6–16/cluster/age). **c** SOD activity according to treatment group (10–13/sex/group/age). **d** SOD activity according to cluster (*n* = 11–17/cluster/age). **e** MDA activity (relative to blank) according to treatment group (*n* = 9–12/sex/group/age). **f** MDA activity (relative to blank) according to cluster (*n* = 8–17/cluster/age). PIC=poly(I:C), PIC-RES=resilient poly(I:C) offspring (CL1), PIC-VULN=vulnerable poly(I:C) offspring (CL2), VEH=all vehicle offspring, VEH (CL1)=vehicle offspring belonging to typical memory cluster. *p ≤ 0.05, **p < 0.01. Data are presented as mean ± SEM.

We next proceeded to analyse plasma biomarkers by treatment group (Fig. 2a, c, e) or cluster membership (Fig. 2b, d, f). There were no effects of treatment group or sex on plasma IL-6 during adolescence, however, when split by sex, male poly(I:C) offspring exhibited elevated plasma IL-6 in contrast to male vehicle controls in adulthood (Fig. 2a; F_1,7.03_ = 5.564, *p* = 0.050). When analysed according to cluster membership, however, there were no significant differences in plasma IL-6 between vulnerable (CL2) or resilient (CL1) clusters during adolescence or adulthood (Fig. 2b).

By contrast, there was a main effect of treatment group on SOD activity during adolescence (Fig. 2c; F_1,47_ = 5.687, *p* = 0.021), with poly(I:C) offspring showing lower plasma SOD activity than vehicle controls. There was additionally a main effect of sex on SOD activity at this developmental stage (F_1,42.145_ = 4.319, *p* = 0.044), with activity levels higher in females than in males. While there was no main effect of sex on plasma SOD activity during adulthood, when split by group, vehicle females exhibited significantly elevated plasma SOD activity in contrast to vehicle males (Fig. 2c; F_1,13.547_ = 12.007, *p* = 0.004). However, there was no difference in SOD activity between poly(I:C) females or males, and there was no effect of treatment group on plasma SOD activity during adulthood (Fig. 2c).

However, when analysed according to cluster membership, adolescent poly(I:C) offspring belonging to the deficit NOR cluster (PIC-VULN (CL2)) exhibited reduced plasma SOD compared to offspring belonging to the typical NOR cluster (Fig. 2d; VEH (CL1) + PIC-RES (CL1); F_1,38_ = 6.003, *p* = 0.019). During adulthood, vulnerable poly(I:C) offspring (PIC-VULN) showed reduced SOD activity in contrast to resilient poly(I:C) offspring (PIC-RES) (Fig. 2d; F_1,22_ = 4.858, *p* = 0.038). There were, however, no effects of treatment group, sex, or cluster membership on relative plasma MDA activity during adolescence or adulthood (Fig. 2e, f).

### Oxidative stress markers are not altered within the hippocampus of resilient or vulnerable offspring

Following evidence for decreased SOD activity within the plasma of vulnerable poly(I:C) offspring, we sought to investigate whether redox balance was also dysregulated within the hippocampus of these offspring.

**Fig. 3:**
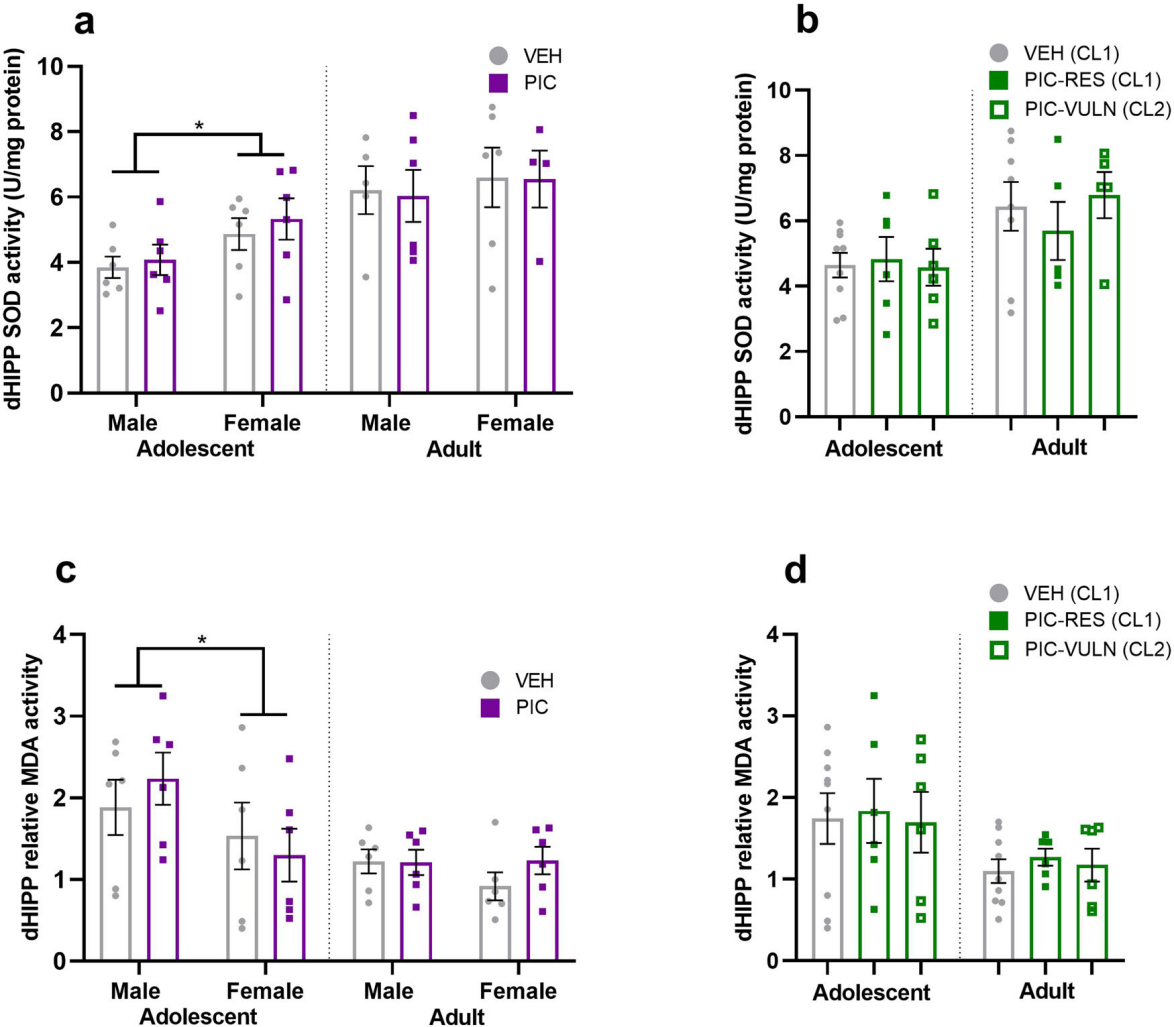
Offspring oxidative stress marker activity in the dorsal hippocampus according to treatment group and cluster membership during adolescence and adulthood. **a** SOD activity according to treatment group (4–6/sex/group/age). **b** SOD activity according to cluster (*n* = 5–9/cluster/age). **c** MDA activity (relative to blank) according to treatment group (*n* = 6/sex/group/age). **d** MDA activity (relative to blank) according to cluster (*n* = 6–9/cluster/age). PIC=poly(I:C), PIC-RES=resilient poly(I:C) offspring (CL1), PIC-VULN=vulnerable poly(I:C) offspring (CL2), VEH=all vehicle offspring, VEH (CL1)=vehicle offspring belonging to typical memory cluster. *p < 0.05. Data are presented as mean ± SEM.

In accordance with the plasma data, there were sex differences in redox balance at adolescence in the hippocampus, with significantly elevated SOD activity (Fig. 3a; F_1,16.62_ = 6.386, *p* = 0.022) and reduced relative MDA activity (Fig. 3c; F_1,13.52_ = 6.743, *p* = 0.022) in female hippocampi. However, there were no effects of treatment group on the activity of SOD or MDA in the adult hippocampus.

While reduced plasma SOD activity predicted vulnerability to MIA-induced deficits (Fig. 2d), there were no significant differences in SOD activity in the hippocampus between resilient or vulnerable clusters (Fig. 3b). In addition, relative MDA activity was not predicted by cluster group (Fig. 3).

## Discussion

The aim of this study was to understand if oxidative stress and the inflammatory profile of MIA offspring could predict MIA-induced cognitive deficits.

First, we confirmed our previous results showing that offspring exposed to poly(I:C)-induced MIA at GD15 exhibit reduced performance in the NOR task during adolescence [13]. While the reduction in NOR performance did not reach significance in adulthood, distinct clustering of offspring into two groups using NOR phenotype allowed us to determine which offspring were resilient and vulnerable to cognitive impairment at both ages. Poly(I:C) significantly affected the distribution to clusters during adulthood, but not during adolescence. While a greater proportion of poly(I:C) offspring belonged to the deficit memory group (CL2) compared to vehicle controls at both stages, 40% of vehicle offspring exhibited a deficit at adolescence in the deficit memory (CL2) cluster compared to only 25% at adulthood. This is surprising considering the evidence for early novel object recognition memory in rats [42, 43], which would suggest a smaller proportion of vehicle animals in the adolescent deficit group. Handling or other methodological differences between studies may explain the discrepancies [44–46]. Nevertheless, behavioural stratification allowed us to investigate brain and blood biomarkers according to cognitive phenotype across the developmental trajectory from adolescence to adulthood.

Recent studies have suggested that redox dysregulation is a major convergence point in the pathogenesis of SCZ, initiated by a number of genetic or environmental factors [16]. SOD functions as an antioxidant by shielding the body from reactive oxygen species (ROS)-related damage and has been suggested to be a target for the treatment of a number of diseases including cancer, diabetes and neurological disorders [47]. In our study, poly(I:C) offspring which exhibited a deficit in NOR (PIC-VULN (CL2)) showed a reduction in plasma but not hippocampal SOD activity, in contrast to all offspring with intact NOR memory (VEH (CL1) and PIC-RES (CL1)) during adolescence. At adulthood, plasma SOD activity in vulnerable poly(I:C) offspring was significantly reduced compared with resilient poly(I:C) offspring only (PIC-RES (CL1)). Thus, a reduction in plasma SOD activity corresponded with MIA-induced cognitive deficits. It remains unclear whether the reduction in plasma SOD directly contributes to the novel object recognition (NOR) deficit or simply reflects broader redox dysregulation within the brain, leading to memory impairment. Surprisingly, SOD activity in the hippocampus did not show any differences between cognitive subtypes. While this suggests that reduced plasma SOD may not be indicative of reduced antioxidant activity in the brain, there are a number of possible explanations why plasma and brain SOD activity do not align in our study. First, due to experimental constraints, the sample size was much larger for plasma than hippocampal studies (11–17 vs 5–9 per cluster, respectively). By increasing the sample size, a clearer distinction in hippocampal SOD activity between clusters may become apparent. In addition, while the hippocampus is an area largely implicated in SCZ pathology [48, 49], studying redox regulation within multiple brain regions, including the prefrontal cortex, as well as more defined regions of the dorsal hippocampus, such as the CA1 region, would be beneficial. Indeed, studies from post-mortem data and SCZ animal models suggest SOD may be reduced in prefrontal regions [50, 51]. However, interestingly, a similar disconnect in SOD activity between plasma and brain tissue was observed in a study that investigated the effect of age on antioxidant function. In this study, aged rats exhibited reduced plasma antioxidant activity including SOD, catalase (CAT) and glutathione peroxidase (GPx), whereas in the brain CAT, GPx, but not SOD activity, were significantly reduced [52]. Together, this data highlights the requirement to investigate alternative antioxidant markers within both the plasma and multiple brain regions of poly(I:C)-exposed offspring.

Nevertheless, previous data shows knockout of extracellular SOD impairs NOR performance in mice [27]. Reduced SOD within the periphery leads to elevated systemic superoxide levels, driving oxidative stress which both activates immune signalling and can compromise blood-brain barrier integrity by damaging endothelial cells [53]. This increased permeability can allow peripheral inflammatory mediators to enter the brain, further exacerbating neuroinflammation [54]. This may trigger a self-perpetuating cycle of neuroinflammation, redox dysregulation, and mitochondrial dysfunction, ultimately damaging neuronal structures, particularly synapses and dendrites, which are critical for memory function, including object recognition [16, 55, 56]. Thus, redox dysregulation within both the brain or periphery could be causative of impaired NOR performance. In fact, superoxide production within the brain is correlated with poor NOR performance in aged mice [28].

Recently, an integrated machine learning approach showed that SOD activity within the blood was the most informative biomarker used to discriminate patients with SCZ from healthy controls [57]. Indeed, numerous studies have demonstrated reduced plasma SOD in patients with SCZ [58–60]. Furthermore, when stratified by clinical subtype, reduced plasma SOD activity has been particularly associated with impaired cognition in SCZ [61–63]. For example, reduced SOD activity in early-onset SCZ patients was significantly correlated with slower information processing speed but not visual learning [58]. A separate study observed a negative correlation between plasma SOD activity and cognitive performance in language and immediate memory tasks, the latter of which may be partially translatable to visual recognition memory [64]. These findings suggest that reduced plasma SOD activity could serve as a potential biomarker for maternal immune activation (MIA)-induced cognitive deficits in SCZ. However, given the broader dysregulation of oxidative stress markers in SCZ, it is likely that an imbalance of multiple redox markers, rather than SOD alone, would provide a more reliable biomarker cognitive impairment associated with schizophrenia.

In support of previous studies [65–68], we detected sex differences in oxidative stress markers in both the plasma and brain. This was particularly prominent at adolescence, with females showing evidence for reduced oxidative stress, demonstrated by increased hippocampal and plasma SOD activity as well as decreased hippocampal activity of MDA. However, at adulthood, a sex difference was only observed in vehicle offspring plasma, again with females exhibiting elevated SOD activity in contrast to males. Indeed, sex hormones have repeatedly been reported to affect redox regulators, representing a potential mechanism underlying sex differences during adolescence [69]. Further research investigating how these sex differences contribute to sex-dependent effects of MIA are essential to better understand the mechanistic pathways underlying the sex-bias observed in NDDs [68, 70, 71].

Given the hypothesised key involvement of IL-6 in both the aetiology of SCZ [72, 73] and as a major pro-inflammatory cytokine of the MIA response [31], we also investigated whether plasma IL-6 was predictive of cognitive phenotype. Despite an increase in plasma IL-6 concentration in poly(I:C) males during adulthood, IL-6 was not predictive of cognitive phenotype, with resilient and vulnerable poly(I:C) offspring exhibiting similar concentrations. Elevated plasma IL-6 in poly(I:C) males may provide evidence in support of the immune priming hypothesis, which postulates that MIA causes a long-term dysregulation to immune cells [74]. However, plasma IL-6 concentration does not appear to correspond with MIA-induced cognitive deficits. A recent study by Mueller et al. [14] showed that poly(I:C)-induced MIA offspring with elevated plasma pro-inflammatory cytokine status showed significant impairments in social behaviour in contrast to MIA offspring with a low plasma pro-inflammatory cytokine status [14]. However, interestingly, cytokine status was not predictive of memory phenotype. This, together with our results, suggests inflammatory plasma biomarkers may be predictive of SCZ subtypes, rather than the symptomology of all disease traits. Indeed, subtypes of patients with SCZ can be significantly predicted by pro-inflammatory cytokine expression, such as in patients with primary and enduring negative symptoms [75, 76]. Future research investigating the differential expression of both plasma pro-inflammatory and anti-inflammatory cytokines in MIA-induced behavioural subtypes, particularly prior to symptom presentation, may be essential in the identification of suitable biomarkers for SCZ.

Our results support further research into the activity of enzymes that contribute to redox balance as potential blood biomarkers for MIA-induced behavioural deficits, with the aim of being able to better predict and treat SCZ. While we have identified a potential biomarker (plasma SOD activity), a longitudinal study sampling blood along the developmental trajectory is essential to understand if this biomarker could predict vulnerability to MIA prior to symptom presentation, and to determine the developmental phase over which this predictive capacity prevails. Furthermore, other factors in the redox response, such as additional antioxidant enzymes as well as markers of oxidative stress must be explored within both the blood and brain across multiple independent cohorts to gain a more complete mechanistic understanding of how redox changes and imbalances can predict behavioural phenotype. By measuring these additional factors and integrating them with SOD levels, the sensitivity and predictive accuracy of redox markers for cognitive impairment in SCZ could be improved. While both sexes were included in this study, sample sizes were not sufficiently powered to investigate plasma biomarker activity within clusters according to sex. The predictive power of individual biomarkers may well differ between male and females given the sex differences in antioxidant response [67], as well sex-dependent variation in MIA-induced brain and behavioural phenotype [70, 71]. Finally, our study only investigated the association between biomarkers and performance in the NOR task, which recapitulates just one aspect of cognitive dysfunction within SCZ [77]. Future work should investigate the expression of brain and blood biomarkers in relation to several behavioural phenotypes following poly(I:C)-induced MIA. Identification of suitable biomarkers for NDDs is a critical step to better guide clinical intervention and personalised medicine approaches [78].

## Supplementary information

Supplementary Material

## Data Availability

Data will be made available upon request.
