# Supplementary material for: Association between redox dysregulation and vulnerability to cognitive deficits induced by maternal immune activation
Source: Transl Psychiatry. 2025 May 26;15:184. doi: 10.1038/s41398-025-03398-0 (PMC12106723; doi:10.1038/s41398-025-03398-0)
Supplement: Supplementary file 1 — Supplementary Material [file 41398_2025_3398_MOESM1_ESM.doc]

**Supplement 1. Maternal and litter effects**

Dams administered with poly(I:C) showed significantly higher concentration of plasma IL-6 3 h post-injection in contrast to vehicle control dams (Supplementary Fig. 1a; t(13)=7.786, *p*<0.001). At PD1, there were no differences in litter size (Supplementary Fig. 1b; t(14)=0.62, *p*=0.868) or pup weight between vehicle control and poly(I:C) litters (Supplementary Fig. 1c; F1,235=2.162, *p=*0.1428). However, as expected, male pups were significantly heavier than female pups (Supplementary Fig. 1c; F1,235=11.94, *p*<0.001). Furthermore, poly(I:C) litters contained a significantly higher proportion of males per litter than vehicle controls (Supplementary Fig. 1d; t(14)=2.648, *p*=0.0191).

**Supplementary figure 1. Maternal and litter effects.** A) Maternal plasma IL-6 concentration IL-6 (n=7-8/group). B) Litter sizes (n=8/group). C) Pup weight at PD1 (n=50-68/sex/group). D) Percentage of males per litter (n=8/group). PIC=poly(I:C), VEH=vehicle. *p<0.05, ***p<0.001. Data are presented as mean ± SEM.

**Supplement 2. Novel object recognition (NOR) test validations and results**

During the acquisition phase of the NOR test, there were no main effects of sex (RM-GLM: F_1,48_=2.556, *p*=0.116) or group (RM-GLM: F_1,48_=0.407, *p*=0.527) on raw exploration of left or right object during adolescence. Similarly, during the acquisition phase in adulthood, there were no main effects of sex (RM-GLM: F_1,43_=0.005, *p*=0.945) or group (RM-GLM: F_1,43_=1.258, *p*=0.268) on raw exploration of left or right object. Paired t-tests showed that exploration of the object in the left or right position did not differ in offspring within the adolescent vehicle (t(25)=1.019, *p*=0.318), adolescent poly(I:C) (t(25)=0.161, *p*=0.873), adult vehicle (t(22)=0.917, *p*=0.369) and adult poly(I:C) (t(23)=1.511, *p*=0.144) groups (Supplementary Fig. 2)

Similarly, although there was a significant reduction in NOR DI between groups during adolescence, when raw exploratory times were compared, group or sex did not affect the exploration of novel versus familiar object during adolescence (RM-GLM: group: F_1,48_=2.121, *p*=0.152, sex: F_1,48_=2.793, *p*=0.101) or adulthood (RM-GLM: group: F_1,40_=1.352, *p*=0.252, sex: F_1,40_=0.117, *p*=0.734). Paired t-tests showed that adolescent vehicle (t(25)=8.348, *p*<0.001), adolescent poly(I:C) (t(25)=3.51, *p*=0.002), adult vehicle (t(22)=3.869, *p*<0.001) and adult poly(I:C) (t(23)=3.506, *p*=0.002) all showed a preference for the novel over familiar object (Supplementary Fig. 2b).

There was no main effects of group or sex on total object exploration between groups during the acquisition phase in adolescence (group: F_1,11.46_=0.318, *p*=0.584, sex: F_1,35.67_=2.958, *p*=0.094) or adulthood (group: F_1,11.374_=0.96, *p*=0.348, sex: F_1,29.404_=0.044, *p*=0.836; Supplementary Fig. 2c). Additionally, total object exploration did not differ between groups or sex during the retention phase during adolescence (group: F_1,18.826_=1.572, *p*=0.225, sex: F_1,41.794_=3.036, *p*=0.089) or adulthood (group: F_1,13.77_=0.117, *p*=0.737, sex: F_1,33.9_=0.39, *p*=0.537; Supplementary Fig. 2d).

**Supplementary figure 2. Raw object exploration times.** A) Raw exploration of the left and right object during the acquisition phase. B) Raw exploration of the novel and familiar object during the retention phase. C) Total object exploration measured in seconds during the acquisition phase. D) Total object exploration in seconds during the retention phase. ACQ=acquisition phase, PIC=poly(I:C), RET=retention phase, VEH=vehicle. **p<0.01, ***p<0.001. Data are presented as mean ± SEM (n=23-26/group/age).

**Supplement 3. Distribution of clusters within each vehicle and poly(I:C) litter**

**Supplementary figure 3. Cluster litter distribution.** CL1=typical memory cluster, CL2=deficit memory cluster. P=poly(I:C), V=vehicle.
